# Crucial control measures to contain China's first Delta variant outbreak

**DOI:** 10.1093/nsr/nwac004

**Published:** 2022-01-18

**Authors:** Lei Luo, Zifeng Yang, Jingyi Liang, Yu Ma, Hui Wang, Chitin Hon, Mei Jiang, Zhengshi Lin, Wenda Guan, Zhitong Mai, Yongming Li, Kailin Mai, Zhiqi Zeng, Chuanmeizi Tu, Jian Song, Bin Liu, Yong Liu, Jianfeng He, Huiyuan Li, Bosheng Li, Hang Dong, Yutian Miao, Shujun Fan, Lirui Fan, Xingyi Liang, Ke Li, Chun Chen, Huihong Deng, Zhicong Yang, Nanshan Zhong

**Affiliations:** Institute of Public Health, Guangzhou Medical University & Guangzhou Center for Disease Control and Prevention, Guangzhou 510440, China; State Key Laboratory of Respiratory Disease, National Clinical Research Center for Respiratory Disease, Guangzhou Institute of Respiratory Health, the First Affiliated Hospital of Guangzhou Medical University, Guangzhou 510120, China; Guangzhou Laboratory, Bio-Island, Guangzhou 510320, China; Guangzhou Key Laboratory for Clinical Rapid Diagnosis and Early Warning of Infectious Diseases, Guangzhou 51000, China; State Key Laboratory of Quality Research in Chinese Medicine, Macau Institute for Applied Research in Medicine and Health, Macau University of Science and Technology, Taipa 999078, China; State Key Laboratory of Respiratory Disease, National Clinical Research Center for Respiratory Disease, Guangzhou Institute of Respiratory Health, the First Affiliated Hospital of Guangzhou Medical University, Guangzhou 510120, China; Guangzhou Laboratory, Bio-Island, Guangzhou 510320, China; Macau Institute of Systems Engineering, Macau University of Science and Technology, Taipa 999078, China; Institute of Public Health, Guangzhou Medical University & Guangzhou Center for Disease Control and Prevention, Guangzhou 510440, China; Institute of Public Health, Guangzhou Medical University & Guangzhou Center for Disease Control and Prevention, Guangzhou 510440, China; Macau Institute of Systems Engineering, Macau University of Science and Technology, Taipa 999078, China; State Key Laboratory of Respiratory Disease, National Clinical Research Center for Respiratory Disease, Guangzhou Institute of Respiratory Health, the First Affiliated Hospital of Guangzhou Medical University, Guangzhou 510120, China; State Key Laboratory of Respiratory Disease, National Clinical Research Center for Respiratory Disease, Guangzhou Institute of Respiratory Health, the First Affiliated Hospital of Guangzhou Medical University, Guangzhou 510120, China; Guangzhou Laboratory, Bio-Island, Guangzhou 510320, China; State Key Laboratory of Respiratory Disease, National Clinical Research Center for Respiratory Disease, Guangzhou Institute of Respiratory Health, the First Affiliated Hospital of Guangzhou Medical University, Guangzhou 510120, China; Guangzhou Laboratory, Bio-Island, Guangzhou 510320, China; Guangzhou Key Laboratory for Clinical Rapid Diagnosis and Early Warning of Infectious Diseases, Guangzhou 51000, China; State Key Laboratory of Respiratory Disease, National Clinical Research Center for Respiratory Disease, Guangzhou Institute of Respiratory Health, the First Affiliated Hospital of Guangzhou Medical University, Guangzhou 510120, China; Guangzhou Laboratory, Bio-Island, Guangzhou 510320, China; State Key Laboratory of Quality Research in Chinese Medicine, Macau Institute for Applied Research in Medicine and Health, Macau University of Science and Technology, Taipa 999078, China; State Key Laboratory of Respiratory Disease, National Clinical Research Center for Respiratory Disease, Guangzhou Institute of Respiratory Health, the First Affiliated Hospital of Guangzhou Medical University, Guangzhou 510120, China; Guangzhou Laboratory, Bio-Island, Guangzhou 510320, China; State Key Laboratory of Respiratory Disease, National Clinical Research Center for Respiratory Disease, Guangzhou Institute of Respiratory Health, the First Affiliated Hospital of Guangzhou Medical University, Guangzhou 510120, China; Guangzhou Laboratory, Bio-Island, Guangzhou 510320, China; State Key Laboratory of Respiratory Disease, National Clinical Research Center for Respiratory Disease, Guangzhou Institute of Respiratory Health, the First Affiliated Hospital of Guangzhou Medical University, Guangzhou 510120, China; Guangzhou Laboratory, Bio-Island, Guangzhou 510320, China; State Key Laboratory of Respiratory Disease, National Clinical Research Center for Respiratory Disease, Guangzhou Institute of Respiratory Health, the First Affiliated Hospital of Guangzhou Medical University, Guangzhou 510120, China; Guangzhou Laboratory, Bio-Island, Guangzhou 510320, China; State Key Laboratory of Respiratory Disease, National Clinical Research Center for Respiratory Disease, Guangzhou Institute of Respiratory Health, the First Affiliated Hospital of Guangzhou Medical University, Guangzhou 510120, China; Guangzhou Laboratory, Bio-Island, Guangzhou 510320, China; State Key Laboratory of Respiratory Disease, National Clinical Research Center for Respiratory Disease, Guangzhou Institute of Respiratory Health, the First Affiliated Hospital of Guangzhou Medical University, Guangzhou 510120, China; Guangzhou Laboratory, Bio-Island, Guangzhou 510320, China; State Key Laboratory of Respiratory Disease, National Clinical Research Center for Respiratory Disease, Guangzhou Institute of Respiratory Health, the First Affiliated Hospital of Guangzhou Medical University, Guangzhou 510120, China; Kingmed Virology Diagnostic & Translational Center, Guangzhou Kingmed Center for Clinical Laboratory Co., Ltd, Guangzhou 510330, China; Guangdong Center for Disease Control and Prevention, Guangzhou 511430, China; Kingmed Virology Diagnostic & Translational Center, Guangzhou Kingmed Center for Clinical Laboratory Co., Ltd, Guangzhou 510330, China; Guangdong Center for Disease Control and Prevention, Guangzhou 511430, China; Institute of Public Health, Guangzhou Medical University & Guangzhou Center for Disease Control and Prevention, Guangzhou 510440, China; Institute of Public Health, Guangzhou Medical University & Guangzhou Center for Disease Control and Prevention, Guangzhou 510440, China; Institute of Public Health, Guangzhou Medical University & Guangzhou Center for Disease Control and Prevention, Guangzhou 510440, China; Institute of Public Health, Guangzhou Medical University & Guangzhou Center for Disease Control and Prevention, Guangzhou 510440, China; Department of Urban and Rural Planning, Shunde Urban and Rural Planning Information Research Center, Foshan 528300, China; Institute of Public Health, Guangzhou Medical University & Guangzhou Center for Disease Control and Prevention, Guangzhou 510440, China; Institute of Public Health, Guangzhou Medical University & Guangzhou Center for Disease Control and Prevention, Guangzhou 510440, China; Guangdong Center for Disease Control and Prevention, Guangzhou 511430, China; Institute of Public Health, Guangzhou Medical University & Guangzhou Center for Disease Control and Prevention, Guangzhou 510440, China; State Key Laboratory of Respiratory Disease, National Clinical Research Center for Respiratory Disease, Guangzhou Institute of Respiratory Health, the First Affiliated Hospital of Guangzhou Medical University, Guangzhou 510120, China; Guangzhou Laboratory, Bio-Island, Guangzhou 510320, China

**Keywords:** COVID-19, public health, control measure, close contact, mass testing

## Abstract

The SARS-CoV-2 B.1.617.2 (Delta) variant flared up in late May in Guangzhou, China. Transmission characteristics of Delta variant were analysed for 153 confirmed cases and two complete transmission chains with seven generations were fully presented. A rapid transmission occurred in five generations within 10 days. The basic reproduction number (R_0_) was 3.60 (95% confidence interval: 2.50–5.30). After redefining the concept of close contact, the proportion of confirmed cases discovered from close contacts increased from 43% to 100%. With the usage of a yellow health code, the potential exposed individuals were self-motivated to take a nucleic acid test and regained public access with a negative testing result. Facing the massive requirement of screening, novel facilities like makeshift inflatable laboratories were promptly set up as a vital supplement and 17 cases were found, with 1 pre-symptomatic. The dynamic adjustment of these three interventions resulted in the decline of Rt from 5.00 to 1.00 within 9 days. By breaking the transmission chain and eliminating the transmission source through extending the scope of the close-contact tracing, health-code usage and mass testing, the Guangzhou Delta epidemic was effectively contained.

## INTRODUCTION

The COVID-19 pandemic has been wreaking havoc around the world since the beginning of 2020. Severe acute respiratory syndrome coronavirus 2 (SARS-CoV-2) is the culprit [1]. Implementation of mass vaccination against SARS-CoV-2 with non-pharmaceutical control measures has proved to be effective in containing the pandemic development. However, one of the big challenges we are confronting at present is the fast mutation of SARS-CoV-2; some mutated viral variants produce second or third waves of COVID-19 in some countries, making great burdens on public health and society [2,3].

The SARS-CoV-2 Delta (B.1.617.2) Variant of Concern (VOC) was first identified in India in October 2020 and later spread quickly across India [4]. As of 13 July 2021, the World Health Organization reports that at least 111 countries, territories and areas have detected the Delta variant, which is expected to become the dominant one globally in the coming months. The high increase in case incidence is believed to be related to the increased transmissibility of the Delta variant [5]; moreover, studies have shown reduced sensitivity of Delta to antibody neutralization, suggesting that Delta spread may be associated with an immune escape [6–9].

An epidemic caused by Delta variant broke out on 21 May 2021 in Guangzhou, China, which was the first Delta outbreak in the country. Guangzhou is the major economic center in Southern China with a population of ∼15 million. In the early stage of the epidemic, we found out that the epidemic was characterized by spatial clustering, a short incubation period, strong infectivity and rapid transmission. However, conventional interventions carried out in the first COVID-19 epidemic in early 2020,

such as wearing facial masks, social distancing, tracing and testing the close contacts and isolating the confirmed cases, were no longer blocking the transmission as quickly as possible. As a result, we implemented some new intervention measures complementary to those mentioned above. In the end, the epidemic was eliminated within 28 days with a relative small number of confirmed cases and without a whole-city lockdown, such that the impact on society was minimized. Here, we present the Delta epidemiological characteristics and the control measures that we adopted to contain the epidemic.

## RESULTS

### Characteristics of Delta transmission in Guangzhou

Between 21 May and 18 June 2021, 48.8 million throat-swab samples were collected and tested for nucleic acid of SARS-CoV-2, among which 153 cases were identified. The demographic characteristics of the confirmed cases are summarized in Supplementary Table S1. All infections were related to different clustering and a significant number of infections were from households or communities (81.53%) and restaurants (41.27%) (Table 1). The incubation time was short, with a median of 4.00 days interquartile range (IQR: 3.00, 6.00) and the median serial time was 3.00 days (IQR: 2.00, 5.00). Besides, a low average Ct value of 22.62 (standard deviation: ±6.37) was also observed (Table 1). The basic reproduction number (R_0_) was 3.50 (95% confidence interval: 2.00–5.62).

**Table 1.** Characteristics of Delta cases in Guangzhou, China from 21 May to 18 June 2021.

Characteristics	No. (%)	Ct value, mean (SD)
Total	153	/
Incubation period (days)—median (IQR)	4.00 (3.00, 6.00)	/
Serial time (days)—median (IQR)	3.00 (2.00, 5.00)	/
Clustering		
Household/community	81 (52.94)	/
Restaurant	41 (26.80)	/
School/workplace	12 (7.84)	/
Other	19 (12.41)	/
Generation		
First	1 (0.70)	16.50 (/)^a^
Second	2 (1.40)	14.03 (1.67)
Third	15 (10.49)	21.08 (5.49)
Fourth	37 (25.87)	21.46 (6.06)
Fifth	43 (30.07)	24.34 (7.27)
Sixth	43 (30.07)	22.94 (5.68)
Seventh	2 (1.40)	31.72 (8.63)
Total	143^b^	22.62 (6.37)

^a^There was only one patient in this generation. ^b^We excluded 10 cases who were unable to trace their previous and next generations, and proportions of different generations were calculated by dividing the number by 143 instead of 153. IQR, interquartile range; SD, standard deviation; Ct, cycle threshold value.

### Transmission scenario unraveled by in-depth epidemiological investigation

**Figure 1.**
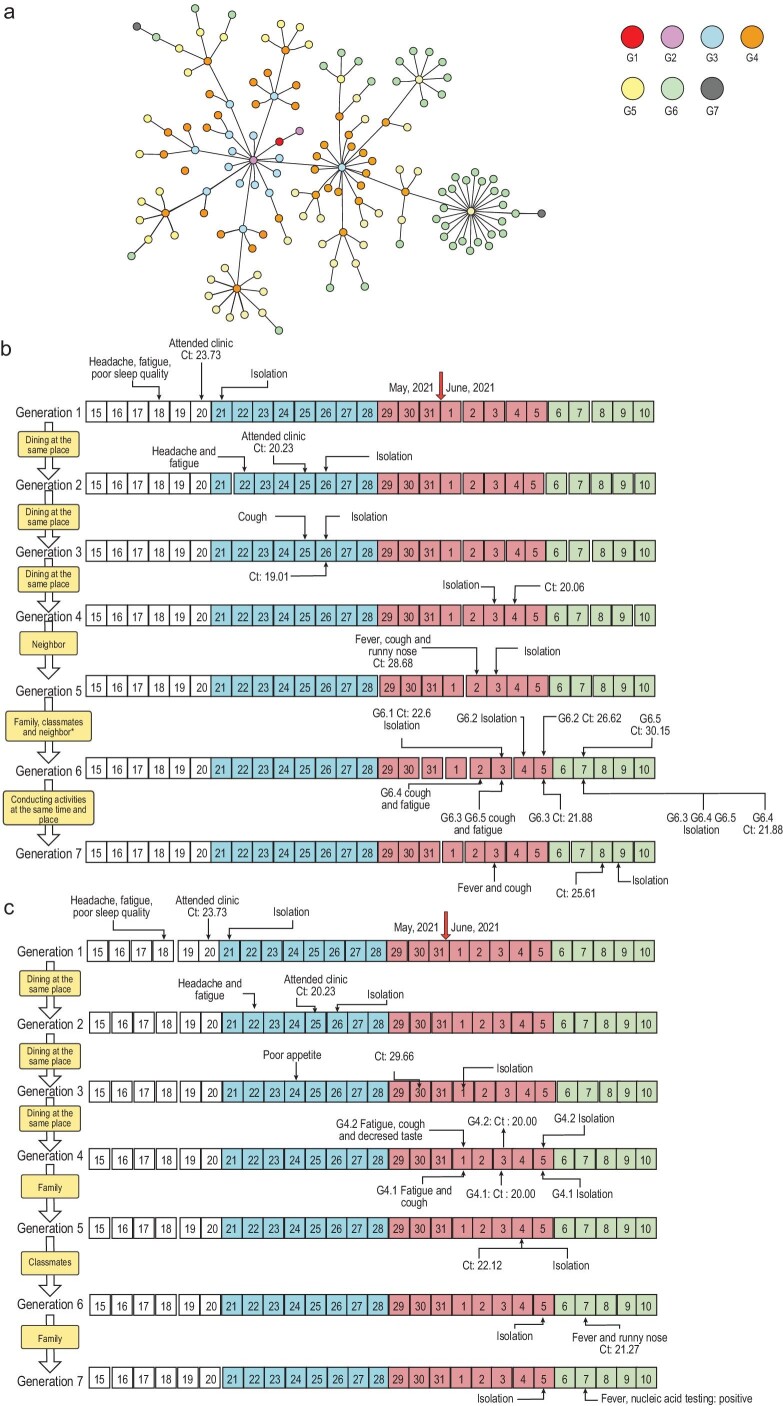
Epidemiological linking network and two transmission chains confirmed by epidemiology investigation and whole-genome sequencing. (a) One hundred and forty-three cases are shown in the network. Cases in each generation are shown in circles with different colors. The first-generation patient (red circle, G1) is in the middle of the network. (b) Transmission Chain I. There are five cases in the sixth generation. G6.1 was a family member of G5, G6.2 was one of the classmates of G6.1. G6.3, G6.2 and G6.1 were the neighbors of G6.4. (c) Transmission Chain II. Highlighted in white represents the time before this epidemic onset; blue, red and green represent the second, third and fourth stages of the epidemic, respectively; the yellow boxes indicate the epidemiological relationships between generations. G, generation of transmission.

Through large-scale and arduous epidemiological investigation with a multipronged approach, among 153 cases with a positive viral nucleic acid test, 143 cases had a clear epidemiological link (Fig. 1a) except for 10 cases with uncertain exposure history. To illustrate how the virus propagated among people, we present two complete transmission chains (seven generations) as examples (Fig. 1b and c). Both transmission chains were initiated with the same patient (Patient G1) of this outbreak, which took place in Liwan District. Patient G1 was a 71-year-old female; she did not feel well on 18 May and visited the fever clinic on 20 May. The diagnosis of SARS-CoV-2 infection was confirmed the next day by a routine nucleic acid test with a Ct value of 23.73. Patient G2 on both transmission chains was the same person, who was not an acquaintance of G1; instead, they dined in the same restaurant, brushed past each other (within several seconds, separated by ∼6 feet) without direct contact. G2 also had a high viral load with a Ct value of 20.23. Thus, the high viral loads of Patients G1 and G2 implied that Delta infectivity was strong. Other cases in Chain I were infected through family and community clustering. Between the earliest date of symptom onset of G2 (22 May) and that of G6 (2 June), a rapid transmission was seen, with the virus spreading through five generations within 10 days (Fig. 1b).

The trajectories of the viral propagation after patient G2 separated. Similar to Chain I, cases on Chain II had a space–time intersection in a restaurant and the virus was further transmitted among family members and classmates over the following days (Fig. 1c). In addition to the close epidemiological relationship, further evidence of phylogenetic analysis of the viral nucleic acid sequences support the cases on each chain were closely related (Supplementary Fig. S1).

### Temporal and spatial distributions of Delta cases across four stages in Guangzhou

**Figure 2.**
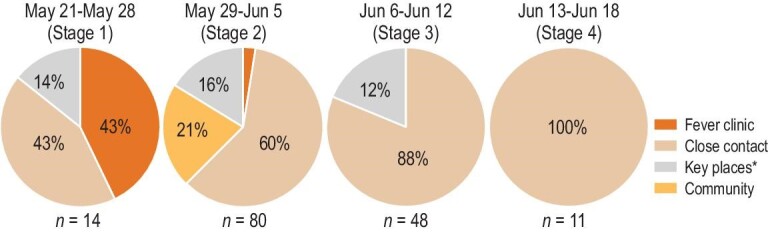
The case-finding source at each stage. Case-finding source includes fever clinic, close contact, key place and community. ^*^Key place refers to where confirmed cases had stayed 4 days before the symptom onset.

This epidemic started in Liwan District and discretely spread over six districts: 127 cases were found in Liwan, 8 cases in Haizhu, 10 cases in Nansha, 4 cases in Panyu, 3 cases in Baiyun and 1 case in Yuexiu. Among the 127 cases in Liwan District, 70.08% (89 cases) were in the Baihedong subdistrict and 23.62% (30 cases) were in the Zhongnan subdistrict. The daily confirmed cases per 10 million people in Liwan District increased from 19.85 before 28 May to 109.15 between 29 May and 5 June, then decreased to 73.71 between 6 June and 12 June, and continued to decline thereafter (Fig. 2).

### Renovated interventions against the Delta variant

#### Close contact redefinition

After realizing the high infectivity of the Delta strains, we therefore redefined close contact to contain those who were potentially at risk of infection. The number of close contact increased from 378 in the first stage to 5992 in the fourth stage (Table 2). We discovered that the confirmed cases were mainly detected through four channels, including the fever clinic, close contact, key places and the community (Fig. 2). With the close-contact redefinition, the proportion of close contact as the source of confirmed cases ultimately increased from 43% to 100% through four stages of the epidemic.

**Table 2.** The key events and public health interventions across the four stages in the campaign against SARS-CoV-2 Delta in Guangzhou.

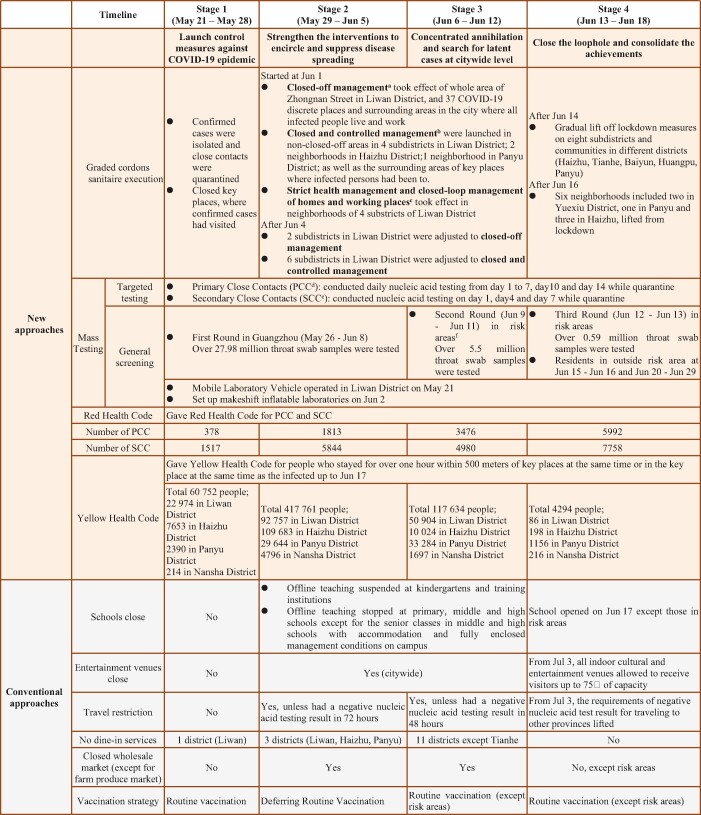

^a^People self-quarantined and performed nucleic acid tests on the 1st to 7th, 10th and 14th days of quarantine. ^b^People could only enter but could not exit their own neighborhood and gatherings were strictly prohibited. Nucleic acid tests were performed on the 1st, 4th and 7th days. ^c^People could only travel between home and working places with negative nucleic acid test results within 24 hours. ^d^Primary close contacts (PCC) referred to people (i) whoever was either in the same space, same workplace or building with infected people; (ii) those who had been with infected people 4 days before disease onset. Disease onset referred to date of symptom onset for symptomatic cases and sampling date of the first positive test result for pre-symptomatic case. ^e^Secondary close contacts (SCC) referred to close contacts of PCC. ^f^Risk areas included places with (i) >50 cumulative number of confirmed cases and at least one cluster was discovered within 14 days; (ii) cumulative number of confirmed non-clustered cases exceeded 50 within 14 days; (iii) newly reported confirmed cases within 14 days.

#### Epidemic control with health codes

**Figure 3.**
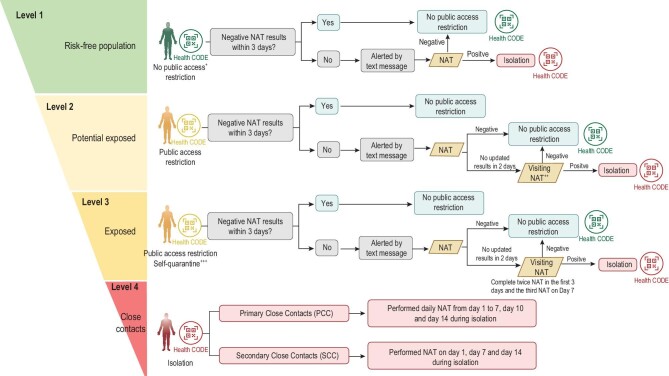
Exposure-risk classification and graded management. **^+^**Public access included public transportation usage and public places entrance. **^++^**Visiting NAT refers to door-to-door sampling process conducted by three-people groups. **^+++^**The exposed individuals were suggested to quarantine at home. NAT, nucleic acid test.

After extending the scope of close-contact tracing, the conduction of epidemiological investigation slowed down due to limited manpower. Thus, people with exposure risk were classified and differentially managed (Fig. 3). In total, 600 441 potential exposed and exposed were identified and labeled with a yellow health code according to case investigation and exposure-risk classification. Individuals assigned a yellow code were considered as having been potentially exposed or exposed to positive cases, and could not access public places; this prompted them to take a nucleic acid test on their own initiative. As long as these people obtained negative nucleic acid test results, they returned to usual life with restrictions lifted off. Among all the identified potential exposed and exposed, a couple with a yellow code participated in community screening test with one of their family members; they all later had a confirmed COVID-19 diagnosis and became the first three cases in Nansha District. After they were isolated, four family members and three close contacts of them were subsequently confirmed with Delta virus infection and isolated promptly, including one pre-symptomatic case (Supplementary Fig. S2). Except for this cluster, no more cases were found in Nansha District. Thus, the progress of the COVID-19 outbreak in Nansha District was quickly contained.

#### Mass testing

With the range of the potential risk population extended, the previously routine nucleic acid testing capacity could not reach our requirement. In addition to the testing volume composed of those from centers for disease control (CDC), hospital and third-party laboratories, novel facilities and makeshift inflatable laboratories were established in several gymnasiums. The maximum daily testing number of these labs increased from 120 000 to 180 000 and samples were received around the clock and issued results within 6 to 10 hours; this manipulation significantly improved the speed of screening. Besides, the mobile testing vehicles were also introduced in risk areas such as Liwan District to save the sample transporting time and restrict the people at risk from moving around. With the joint effort of various institutes, the cumulative testing number reached 48.80 million samples within a month, including 34.07 million samples citywide and 14.73 million samples from risk areas (Supplementary Fig. S3). Seventeen positive cases outside

the risk areas were found through mass testing including one pre-symptomatic case.

#### Effectiveness of epidemic control

**Figure 4.**
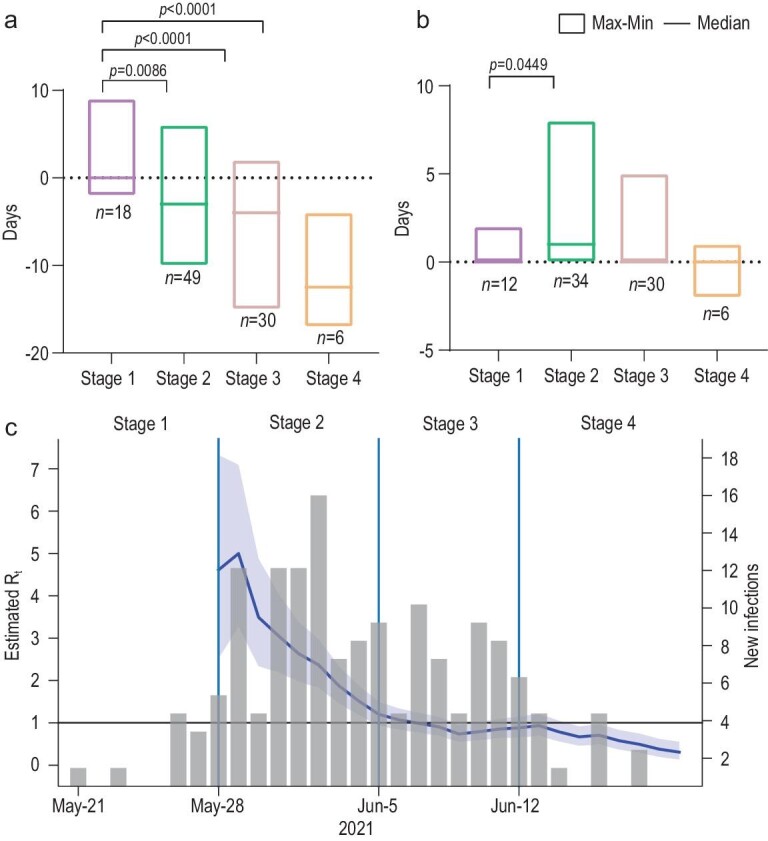
Effectiveness of epidemic control. (a) Interval between symptom onset and isolation among close contacts. (b) Interval between close-contacts identification and isolation. (c) Rt throughout the epidemic. Gray columns represent daily reported cases. The blue line represents the dynamic change of Rt and the surrounding purple area refers to 95% CI of Rt. The light-blue vertical lines separate the epidemic course into four stages.

With the facilitation of new approaches, the median interval between symptoms onset and isolation shortened from 0.44 to –11.50 days on average (Fig. 4a), indicating that the infected cases had been isolated even before symptom onset. Moreover, the interval between close contacts identified and isolation shortened from 2 days at the early stages to <1 day at the end (Fig. 4b). These data suggested that the infected cases were under control before the symptom onset, so the transmission risk was cut off promptly and effectively. Although the effective reproduction number (Rt) sharply increased at the beginning of the second stage with a peak of 5.00 on 29 May, it quickly declined subsequently and was <1.00 on 7 June 2021. In total, cases of COVID-19 and the Rt were reduced effectively along with the four-stage intervention (Table 2 and Fig. 4c).

## DISCUSSION

The SARS-CoV-2 Delta variant has rapidly spread worldwide, becoming the dominant epidemic strain. The epidemiology of Delta strain in Guangzhou is characterized by spatial clustering, a short incubation period, strong infectivity and rapid transmission. With close-contact redefinition, the implementation of a health code and mass testing, plus routine control measures, the time taken to isolate confirmed cases and Rt have rapidly declined. Within 28 days, the first Delta outbreak in China was contained.

In light of 153 confirmed cases, a majority of them showed a strong relation to the same space, mainly in restaurants and households. Additionally, the incubation period estimated in the present study is shorter than that in previous studies (4.0 vs.5.2 [10,11]). Besides, an average Ct value of 22.62 is observed, which is lower than that of the SARS-CoV-2 B.1.1.7 lineage reported by Frampton [12] and van Loon W [13]. Moreover, the average serial interval was 3 days, which is consistent with that reported by a previous study [14]. Different from the epidemic caused by the SARS-CoV-2 wild-type strain in Guangzhou last year [15], the strong infectivity of the Delta strain necessitates intervention measures to be modified and more specific and effective.

In the early stage of epidemiological investigation, we considered primary close contact as people living together or having contact at a certain distance (≤2 meters) for >15 minutes with confirmed cases, which is consistent with the definition implemented by the USA [16], UK [17] and Singapore [18]. However, a brush-into-infection case occurred in a public enclosed space, suggesting that possible aerosol transmission could not be overlooked [19]. In view of this situation, we redefined the concept of close contact to include whoever is with an infected person in the same places such as offices, buildings and entertainment venues or those who had been with an infected person 4 days before the disease onset. It is this new strategy that expanded our surveillance scope and controlled the risk of transmission as early as possible. As can be seen in our result, the source of confirmed cases was gradually narrowed into the key surveillance population.

The rapid development and utilization of mobile health applications has been seen since the beginning of COVID-19 in many territories and jurisdictions throughout five continents with government support [20–22]. Up to August 2020, 63 mobile apps had been used, the functionality of which includes contact tracing, self symptom checking, GPS location tracing, exposure notification, awareness raising and information sharing, etc. In our study, by having close contacts redefined, more people with exposure risk need investigation (Table 2). The execution of both targeted tracing of close contacts and comprehensively screening the potential exposed could be a big challenge under limited time and manpower. Therefore, we started to use a mobile app at Stage 2; the stratified health-code designation greatly facilitated and accelerated the contact-tracing process (Table 2 and Figs 3 and 4). Our experience on mobile app applications proves that modern technology is a requisite for epidemic control like other countries do.

To improve the screening speed and ensure testing volume, mass testing was implemented in many countries. Similar to German government action [23], we realized the great importance of expanding testing capacity. Not only hospitals and CDC, but also third-party laboratories have participated in the battle against Delta. Moreover, we applied makeshift inflatable laboratories and mobile testing vehicles synchronously into mass testing, reaching a 180 000 testing volume per day. This strategy also has been dynamically adjusted from citywide to risk areas in order to precisely block the channels of transmission.

In addition to the new measures mentioned above, routine prevention and control measures were executed (Table 2). First, similar to the 2019 Wuhan epidemic [24], we have also imposed restrictions on the movement of people. People leaving Guangdong Province were requested for negative nucleic acid tests and green codes; no confirmed cases so far were found outside the border during this outbreak. Second, quarantine measures were adopted. We not only imposed self-quarantine on people with exposure risk as many countries did [25,26], but also persuaded those with poorer living conditions to go to hotels for centralized quarantine. Third, we temporarily halted the vaccination program to avoid people gathering and false-positive nucleic acid results in mass testing. Currently, animal surveillance is ongoing in regard to the cross-species infection [27,28].

The mutation of SARS-CoV-2 is accelerating, and the control of the pandemic is still challenging. The containment of the Delta epidemic in Guangzhou has taught us several lessons. First of all, because this outbreak was possibly initiated from an imported case, it reminds us that the management of port cities and independent travelers should be strengthened. Second, prevention and control strategies should adhere to current characteristics of the epidemic transmission and improve in accordance with the changing situation of the epidemic. Reasonably classifying risk levels, accurately delimiting control units and taking measures as soon as possible are indispensable. Finally, to strike a good balance between epidemic control and socio-economic development, it is necessary to call for more precise prevention and control measures to control outbreaks.

## CONCLUSION

When a local epidemic breaks out caused by a highly infectious pathogen like SARS-CoV-2 Delta, it is necessary to promptly adjust the prevention and control measures according to the epidemic situation. Precise prevention and control policies help to not only control the epidemic, but also minimize the epidemic impact on society and the economy.

## MATERIALS AND METHODS

### Source of data

Data of the demographics of confirmed cases from 18 May to 18 June 2021 were extracted from the collection of Guangzhou Center for Disease Control and Prevention, including patients’ birth date, sex and residential district. The population size in each stratum was extracted from the Guangzhou 2019 Statistical Yearbook.

### Diagnosis and epidemiological investigation of confirmed case

The collection and detection of nasopharyngeal swabs were performed by strictly following the Prevention and Control Protocols of COVID-19 (Edition 8). Based on open reading frame 1ab (ORF 1ab) and nucleocapsid (N) protein genes in the SARS-CoV-2 genome, RT-PCR was performed according to the manufacturer's specification (Da’An Gene Co., Ltd of Sun Yat-sen University) [29]. If the cycle threshold (Ct) value of RT-PCR is <40, then the sample is considered to be positive. The exposure histories of confirmed cases and their close contacts were obtained through an interview, public video monitoring systems and cell-phone apps, etc. The sequence analysis of cases is shown in supplementary data.

### Renovated measures implementation

- Update the concept of close contact to expand the scope of tracking. The original definition of close contact is family members or colleagues who are with the infected person 2 days before the onset of the disease, or people who had meals or meetings with the infected person within a 1-meter distance. The new concept of close contact for Delta virus was described as: (a) whoever is either in the same space, the same workplace or the same building as the infected person; (b) those who had been with the infected person 4 days before the disease onset.
- Exposure-risk classification and graded management with health code. Based on the possibility of exposure to the source of infection, people with exposure risk were classified and divided into four levels, including (a) risk-free population, (b) potential exposed, (c) exposed and (d) close contacts; definitions of these four groups and the detailed corresponding management measures are shown in Fig. 3.
- Set up makeshift inflatable laboratories. Multiple standardized makeshift inflatable laboratories were built in gymnasiums within 24 hours to increase the testing capacity. Each inflatable laboratory covers an area of ∼210 square meters, which is divided into three areas: reagent preparation area, sample processing area and nucleic acid amplification area (Supplementary Fig. S4).

According to the judgment and prediction on the epidemic trend, we literally divided the whole epidemic into four stages. More detailed control measures in each stage can be found in Table 2.

### Statistical analyses

Normal distribution data such as Ct values were presented as mean (± standard deviation). Qualitative information such as the source of case findings was presented as a percentage or frequency. Analysis of variance was performed in the comparison of interval calculation across four stages. All of the tests were two-tailed and a value of *P* < 0.05 represented statistical significance. The epidemiological parameters (R_0_ and Rt) calculation is shown in supplementary data.

## DATA AVAILABILITY

The data that support the findings of this study originate from Guangzhou Center for Disease Control and Prevention. Case data are derived from epidemiological investigation reports that are not publicly available.

## Supplementary Material

nwac004_Supplemental_File
