# Supplementary material for: Crucial control measures to contain China's first Delta variant outbreak
Source: Natl Sci Rev. 2022 Jan 18;9(4):nwac004. doi: 10.1093/nsr/nwac004 (PMC9046578; doi:10.1093/nsr/nwac004)
Supplement: nwac004_Supplemental_File [file nwac004_supplemental_file.docx]

**Supplementary Data**

**Materials and methods**

**Viral RNA sequencing and analysis**

**1.1 Sample preparation**

Total RNA was extracted from 200 μL of nasopharyngeal swab samples (SARS-CoV-2 nucleic acid test positive) using viral RNA/DNA mini kit (Invitrogen) according to manufacturer`s instruction, resuspended in the RNase-free water; the extracted total RNA was used directly for cDNA synthesis and amplification with ULSEN SARS-CoV-2 whole genome kit (MicroFuture) in accordance with the manufacturer`s manual.

**1.2 Whole Genome Sequencing**

The PCR products were respectively purified with AMPure XP beads (Beckman Coulter Genomics, Danvers, MA) and submitted to library preparation procedure with the Nextera XT DNA Library Preparation Kit (Illumina, San Diego, CA). Sample libraries were loaded onto Miniseq platform (Illumina, San Diego, CA) and 20 M short reads for each sample were generated by 100 cycles single-end sequencing protocol. All sequencing reads after primer trimming and mapped to the reference sequence (the index case, XG5137_GZ_2021/5/21), and the generated consensus sequences were named with GWHBDIM01000000-GWHBDNH01000000. All information has been submitted to the National Genomics Data Center (https://bigd.big.ac.cn/) with submission number CRA004571.

**1.3 Phylogenetic analysis**

Full-length sequences assembly was performed with CLC Genomics Workbench 20, applying a read depth cutoff of ≥15 to the final sequence. Multi sequence alignment was performed by MATFF with other linkage strains obtained from GISAID (https://www.gisaid.org/, Table S3). The phylogenetic tree was built by neighbor-joining method using MAGAX software considering a bootstrap of 1000.

**Estimation of the basic and effective reproduction number (R_0_ and Rt)**

R_0_ is defined as the expected number of additional cases that one case will generate, which was estimated by maximum-likelihood method based on cases with symptoms onset between 18 May and 29 May 2021. We applied a Gamma distribution for the generation time, with a shape parameter (mean: 3.42 days) and a scale parameter (standard deviation: 1.09 days) derived from the epidemiological survey.

We applied a Bayesian framework to estimate the Rt value of Delta strains, which used a Gamma distributed prior, conjugated to the Poisson likelihood, and obtained an analytical formulation of the posterior distribution of Rt. To maintain the accuracy of the prediction and without hiding the underlying time trend, Rt values were estimated over a 7-day moving window.

**Table S1. Demographic Characteristics of SARS-CoV-2 Delta Variant Outbreak in Guangzhou, 2021**

| **Characteristics** | **No. (%)** |
| --- | --- |
| **Total** | 153 |
| **Male—no. (no./total no.%)** | 63 (41.18) |
| **Age(years)—median (IQR)** | 50 (31,67) |
| **Age group(years)—no. (no./total no.%)** |  |
| **<15** | 21 (13.73) |
| **15-44** | 40 (26.14) |
| **45-64** | 48 (31.37) |
| **≥65** | 44 (28.76) |
| **Source—no. (no./total no.%)** |  |
| **Close contact** | 104 (67.97) |
| **Key place** | 24 (15.69) |
| **Community** | 17 (11.11) |
| **Fever clinic** | 8 (5.23) |
| **Case type—no. (no./total no.%)** |  |
| **Pre-symptomatic** | 8 (5.23) |
| **symptomatic** | 145 (94.77) |

| Sequence ID | Percentage of coverage | Number of mutant on nucleic acid | Gender | Age | Area | Ct value | Onset Date | |
| --- | --- | --- | --- | --- | --- | --- | --- | --- |
| CAG1/CBG1 | 99.89% | 35 | Female | 75 | Liwan district | 23.73 | | 18/05/2021 |
| CAG2/CBG2 | 99.87% | 36 | Female | 74 | Liwan district | 20.23 | | 22/05/2021 |
| CAG3 | 99.76% | 37 | Female | 79 | Haizhu district | 29.66 | | 24/05/2021 |
| CAG4 | 99.87% | 37 | Male | 31 | Nansha district | 20 | | 01/06/2021 |
| CAG5 | 99.20% | 38 | Female | 5 | Nansha district | Unknown | | 04/06/2021 |
| CAG6 | 99.96% | 38 | Female | 5 | Nansha district | 21.27 | | 07/06/2021 |
| CAG7 | 81.43% | 30 | Female | 58 | Nansha district | Unknown | | 07/06/2021 |
| CBG3 | 99.76% | 37 | Female | 86 | Liwan district | Unknown | | 25/05/2021 |
| CBG4 | 99.33% | 36 | Female | 82 | Liwan district | Unknown | | 04/06/2021 |
| CBG5 | 99.92% | 36 | Male | 38 | Liwan district | 28.68 | | 02/06/2021 |
| CBG6.1 | 99.41% | 36 | Female | 44 | Liwan district | 30.15 | | 03/06/2021 |
| CBG6.2 | 99.55% | 36 | Male | 81 | Liwan district | 21.88 | | 02/06/2021 |
| CBG6.3 | 99.81% | 36 | Female | 72 | Liwan district | 21.88 | | 03/06/2021 |
| CBG6.4 | 99.73% | 36 | Male | 12 | Liwan district | 26.62 | | 05/06/2021 |
| CBG6.5 | 74.59% | 28 | Male | 12 | Liwan district | 22.60 | | 03/06/2021 |
| CBG7 | 98.87% | 37 | Male | 53 | Liwan district | 25.61 | | 03/06/2021 |

**Table S2. Case information from two transmission chains**

**Table S3. Reference sequence information**

| Lineage | Strains | Accession ID | |
| --- | --- | --- | --- |
| [B.1.617.1](https://ngdc.cncb.ac.cn/ncov/lineage?lineage=B.1.617.1#goto) | [hCoV-19/India/MH-IGIB-GSEQ-1410/2021](https://ngdc.cncb.ac.cn/ncov/genome/accession?q=EPI_ISL_2878849) | | [EPI_ISL_2878849](https://www.gisaid.org/) |
|  | [hCoV-19/India/MH-IGIB-NIV-INSACOG-GSEQ-1418/2021](https://ngdc.cncb.ac.cn/ncov/genome/accession?q=EPI_ISL_2546058) | | [EPI_ISL_2546058](https://www.gisaid.org/) |
| [B.1.617.2](https://ngdc.cncb.ac.cn/ncov/lineage?lineage=B.1.617.2#goto) | [hCoV-19/England/ALDP-182FFB8/2021](https://ngdc.cncb.ac.cn/ncov/genome/accession?q=EPI_ISL_2865326) | | [EPI_ISL_2865326](https://www.gisaid.org/) |
|  | hCoV-19/Scotland/QEUH-15C180B/2021 | | [EPI_ISL_2395413](https://www.gisaid.org/) |
| [B.1.617.3](https://ngdc.cncb.ac.cn/ncov/lineage?lineage=B.1.617.3#goto) | [hCoV-19/India/MH-NCCS-87400/2021](https://ngdc.cncb.ac.cn/ncov/genome/accession?q=EPI_ISL_1415277) | | [EPI_ISL_1415277](https://www.gisaid.org/) |
|  | [hCoV-19/Russia/SPE-RII-32716S/2021](https://ngdc.cncb.ac.cn/ncov/genome/accession?q=EPI_ISL_1797436) | | [EPI_ISL_1797436](https://www.gisaid.org/) |
| [B.1.119](https://ngdc.cncb.ac.cn/ncov/lineage?lineage=B.1.119#goto) | [hCoV-19/USA/IL-NM-0436/2020](https://ngdc.cncb.ac.cn/ncov/genome/accession?q=EPI_ISL_626407) | | [EPI_ISL_626407](https://www.gisaid.org/) |
| [B.1.203](https://ngdc.cncb.ac.cn/ncov/lineage?lineage=B.1.203#goto) | [hCoV-19/Costa Rica/INC-0080/2020](https://ngdc.cncb.ac.cn/ncov/genome/accession?q=EPI_ISL_527748) | | [EPI_ISL_527748](https://www.gisaid.org/) |
| [B.1.342](https://ngdc.cncb.ac.cn/ncov/lineage?lineage=B.1.342#goto) | [hCoV-19/Italy/MAR-AMC-201209279-AN/2020](https://ngdc.cncb.ac.cn/ncov/genome/accession?q=EPI_ISL_2308740) | | [EPI_ISL_2308740](https://www.gisaid.org/) |
| [B.1.415](https://ngdc.cncb.ac.cn/ncov/lineage?lineage=B.1.415#goto) | [SARS-CoV-2/human/USA/WI-UW-780/2020](https://ngdc.cncb.ac.cn/ncov/genome/accession?q=MT795891) | | EPI_ISL_495484 |
| [B.1.513](https://ngdc.cncb.ac.cn/ncov/lineage?lineage=B.1.513#goto) | [hCoV-19/Austria/CeMM0992/2020](https://ngdc.cncb.ac.cn/ncov/genome/accession?q=EPI_ISL_583836) | | [EPI_ISL_583836](https://www.gisaid.org/) |
| A | hCoV-19/Finland/4AS3D428/2020 | | [EPI_ISL_1240950](https://www.gisaid.org/) |
|  | [hCoV-19/Netherlands/UT-RIVM-12697/2021](https://ngdc.cncb.ac.cn/ncov/genome/accession?q=EPI_ISL_1035477) | | [EPI_ISL_1035477](https://www.gisaid.org/) |
| C.1 | hCoV-19/England/MILK-176835E/2021 | | [EPI_ISL_2718062](https://www.gisaid.org/) |
| C.2 | [hCoV-19/South Africa/KRISP-EC-K005318/2020](https://ngdc.cncb.ac.cn/ncov/genome/accession?q=EPI_ISL_678610) | | [EPI_ISL_678610](https://www.gisaid.org/) |
| D.2 | [SARS-CoV-2/human/AUS/VIC11257/2020](https://ngdc.cncb.ac.cn/ncov/genome/accession?q=MW157134) | | EPI_ISL_563311 |
|  | [SARS-CoV-2/human/AUS/VIC9409/2020](https://ngdc.cncb.ac.cn/ncov/genome/accession?q=MW156070) | | EPI_ISL_565539 |
| G.1 | [COG-UK/ALDP-9E68D2](https://ngdc.cncb.ac.cn/ncov/genome/accession?q=OA989876) | | EPI_ISL_580434 |
|  | [hCoV-19/Wales/CAMC-A3E95F/2020](https://ngdc.cncb.ac.cn/ncov/genome/accession?q=EPI_ISL_625449) | | [EPI_ISL_625449](https://www.gisaid.org/) |
| K.1 | hCoV-19/South Korea/KCDC2796/2020 | | [EPI_ISL_526730](https://ngdc.cncb.ac.cn/ncov/23) |
| K.2 | hCoV-19/Russia/KAM-RII-MH12206S/2020 | | [EPI_ISL_872970](https://www.gisaid.org/) |
| L.1 | [hCoV-19/Canada/NS-NML-2241/2020](https://ngdc.cncb.ac.cn/ncov/genome/accession?q=EPI_ISL_915168) | | [EPI_ISL_915168](https://www.gisaid.org/) |
| L.3 | [COG-UK/QEUH-C3327B](https://ngdc.cncb.ac.cn/ncov/genome/accession?q=OD911045) | | EPI_ISL_734123 |
| M.1 | [hCoV-19/Israel/CVL-81-ngs/2020](https://ngdc.cncb.ac.cn/ncov/genome/accession?q=EPI_ISL_776620) | | [EPI_ISL_776620](https://www.gisaid.org/) |
|  | [hCoV-19/Israel/CVL-3290/2021](https://ngdc.cncb.ac.cn/ncov/genome/accession?q=EPI_ISL_1209955) | | [EPI_ISL_1209955](https://www.gisaid.org/) |
| N.1 | [hCoV-19/Suriname/SR-63/2020](https://ngdc.cncb.ac.cn/ncov/genome/accession?q=EPI_ISL_518812) | | [EPI_ISL_518812](https://www.gisaid.org/) |
|  | SARS-CoV-2/human/USA/FL-BPHL-0527/2020 | | EPI_ISL_508730 |
| P.1 | [SARS-CoV-2/human/USA/MN-MDH-6707/2021](https://ngdc.cncb.ac.cn/ncov/genome/accession?q=MZ171518) | | EPI_ISL_2036067 |
| P.3 | hCoV-19/Netherlands/FL-RIVM-24324/2021 | | [EPI_ISL_1597203](https://www.gisaid.org/) |
| R.1 | [hCoV-19/Japan/PG-38479/2021](https://ngdc.cncb.ac.cn/ncov/genome/accession?q=EPI_ISL_2327686) | | [EPI_ISL_2327686](https://www.gisaid.org/) |
| R.2 | SARS-CoV-2/human/USA/RI-Broad_RIDOH-00410/2021 | | EPI_ISL_1253987 |
| S.1 | [hCoV-19/Latvia/137/2020](https://ngdc.cncb.ac.cn/ncov/genome/accession?q=EPI_ISL_639634) | | [EPI_ISL_639634](https://www.gisaid.org/) |
| S.1 | [hCoV-19/Latvia/201/2020](https://ngdc.cncb.ac.cn/ncov/genome/accession?q=EPI_ISL_770034) | | [EPI_ISL_770034](https://www.gisaid.org/) |
| U.1 | COG-UK/ALDP-1006650 | | EPI_ISL_864002 |
| U.3 | hCoV-19/Norway/3028/2021 | | [EPI_ISL_1192319](https://www.gisaid.org/) |
| V.1 | COG-UK/CAMC-A41A92 | | EPI_ISL_822311 |
| V.2 | [hCoV-19/Northern Ireland/NIRE-2356D1/2020](https://ngdc.cncb.ac.cn/ncov/genome/accession?q=EPI_ISL_742130) | | [EPI_ISL_742130](https://www.gisaid.org/) |
| W.1 | [hCoV-19/Portugal/PT2502/2021](https://ngdc.cncb.ac.cn/ncov/genome/accession?q=EPI_ISL_941551) | | [EPI_ISL_912843](https://www.gisaid.org/) |
|  | hCoV-19/Portugal/PT2502/2021 | | [EPI_ISL_941551](https://www.gisaid.org/) |
| Y.1 | [hCoV-19/Portugal/PT1743/2020](https://ngdc.cncb.ac.cn/ncov/genome/accession?q=EPI_ISL_731980) | | [EPI_ISL_731980](https://www.gisaid.org/) |
|  | [hCoV-19/Portugal/PT3464/2021](https://ngdc.cncb.ac.cn/ncov/genome/accession?q=EPI_ISL_1117167) | | [EPI_ISL_1117167](https://www.gisaid.org/) |
| Z.1 | [hCoV-19/Wales/PHWC-4976F9/2020](https://ngdc.cncb.ac.cn/ncov/genome/accession?q=EPI_ISL_726870) | | [EPI_ISL_726870](https://www.gisaid.org/) |
|  | [hCoV-19/Wales/PHWC-49EB5D/2020](https://ngdc.cncb.ac.cn/ncov/genome/accession?q=EPI_ISL_743099) | | [EPI_ISL_743099](https://www.gisaid.org/) |

**
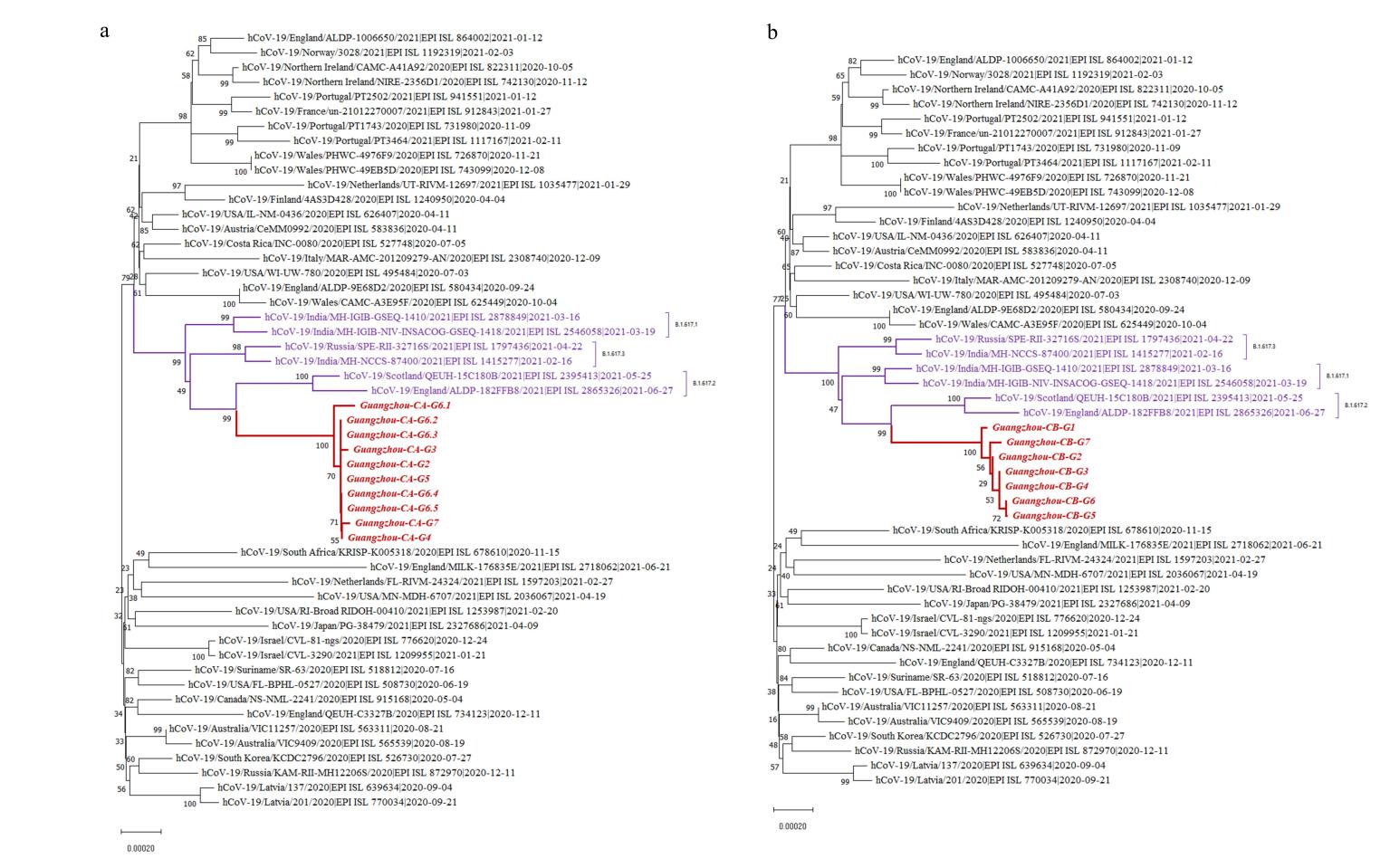
**

**Fig. S1 Phylogenetic analysis of cases in each transmission chain and other SARS-CoV-2 variants. a,** transmission chainⅠ. **b,** transmission chainⅡ. The red branches are Guangzhou cases and the purple branches are B.1.617 variants（B1.617.1，B1.617.2 and B1.617.3）.The phylogenetic tree was built by neighbor-joining method using MAGAX software considering a bootstrap of 1000.

**
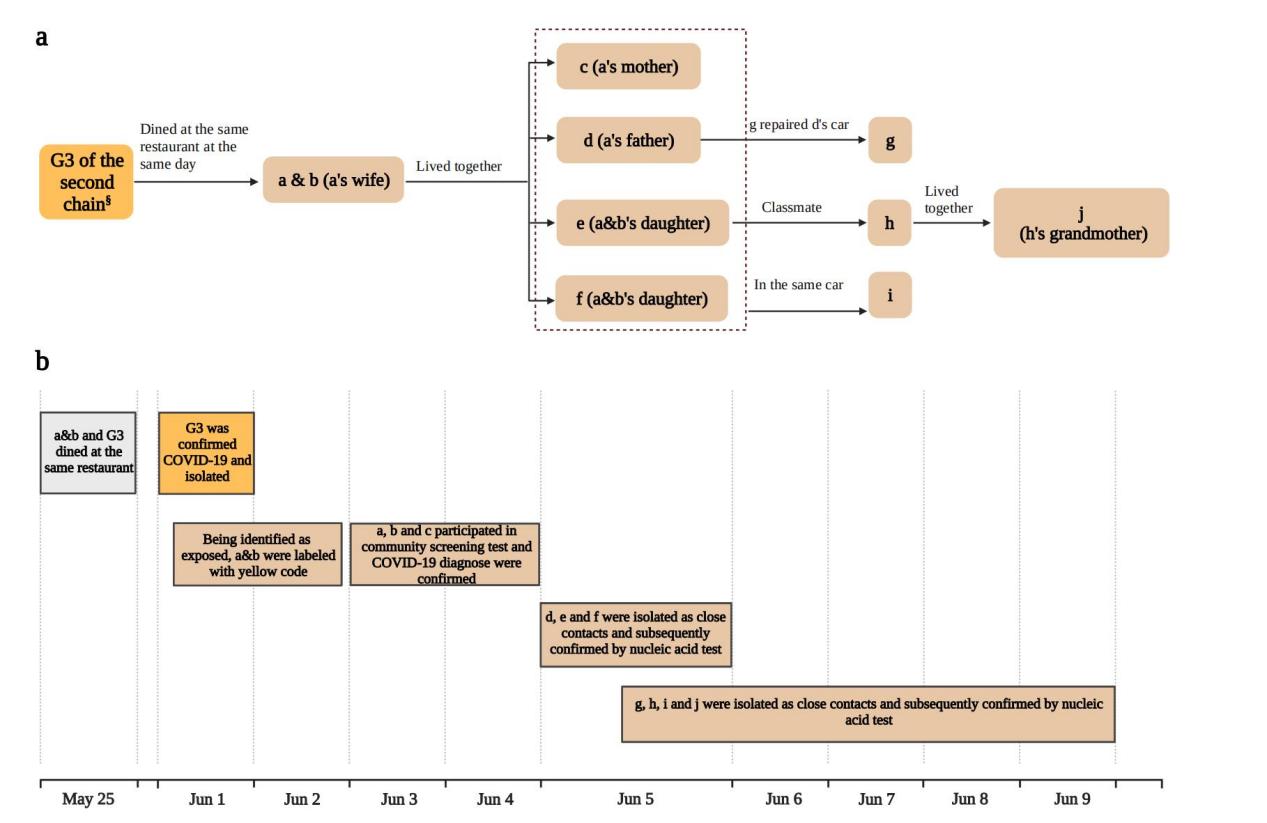
**

**Fig. S2 Transmission chain and discovery timeline in Nansha District**

**a,** Transmission chain in Nansha District. **b,** Discovery timeline of COVID-19 cases in Nansha District, 2021.^§^G3 of transmission chain B (Fig. 1) developed symptoms on May 24, confirmed as COVID-19 and isolated on June 1; a, b, c, d, e, f, g, h, i and j comprised the complete transmission chain in Nansha District

**
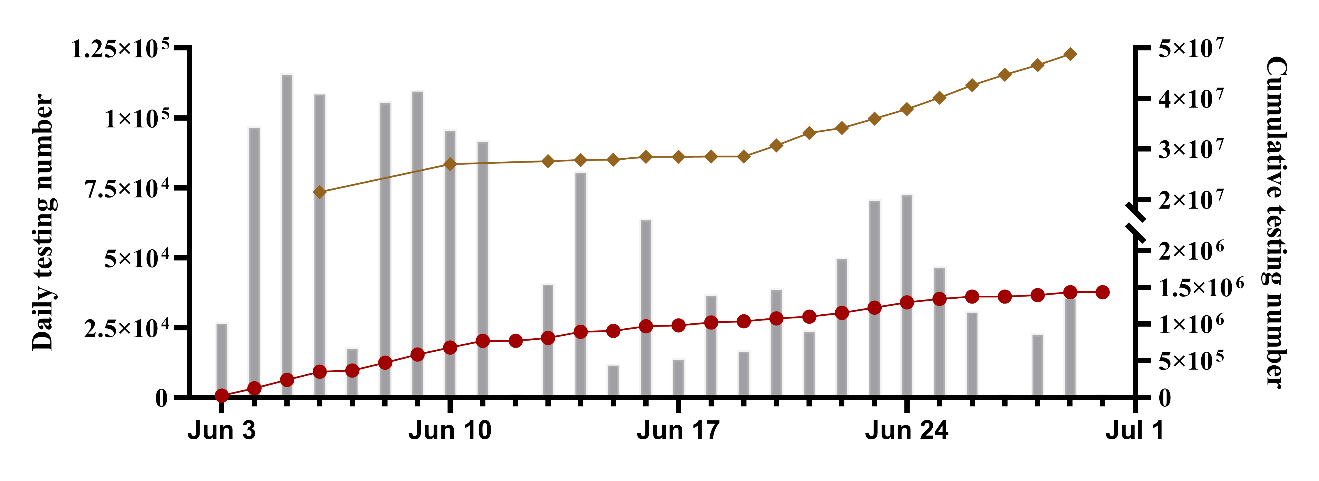
**

**Fig. S3** **Nucleic acid testing volume along the timeline of Delta outbreak**. Brown line refers to cumulative testing number of Guangzhou; Red line refers to cumulative testing number of makeshift inflatable laboratories; grey column refers to daily testing number of makeshift inflatable laboratories.

**a**


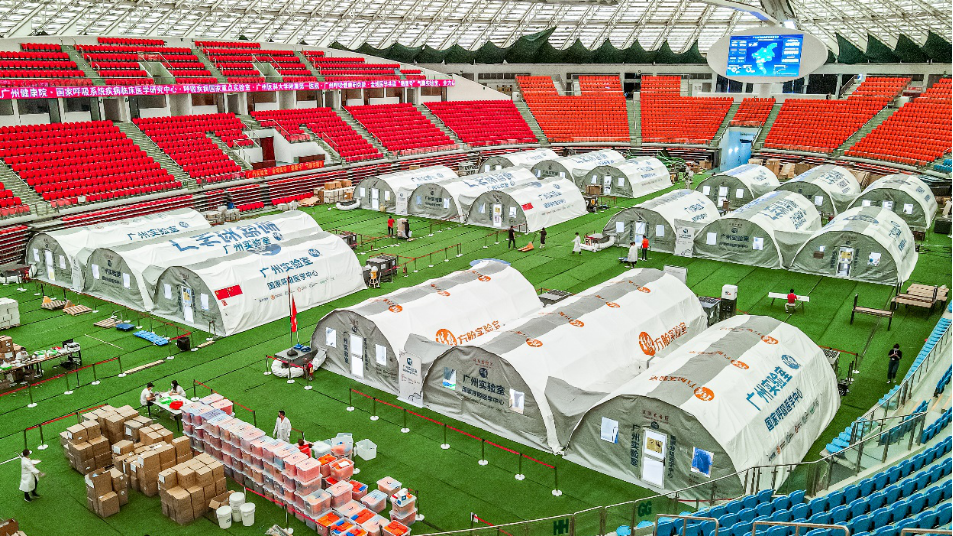


**b**

**
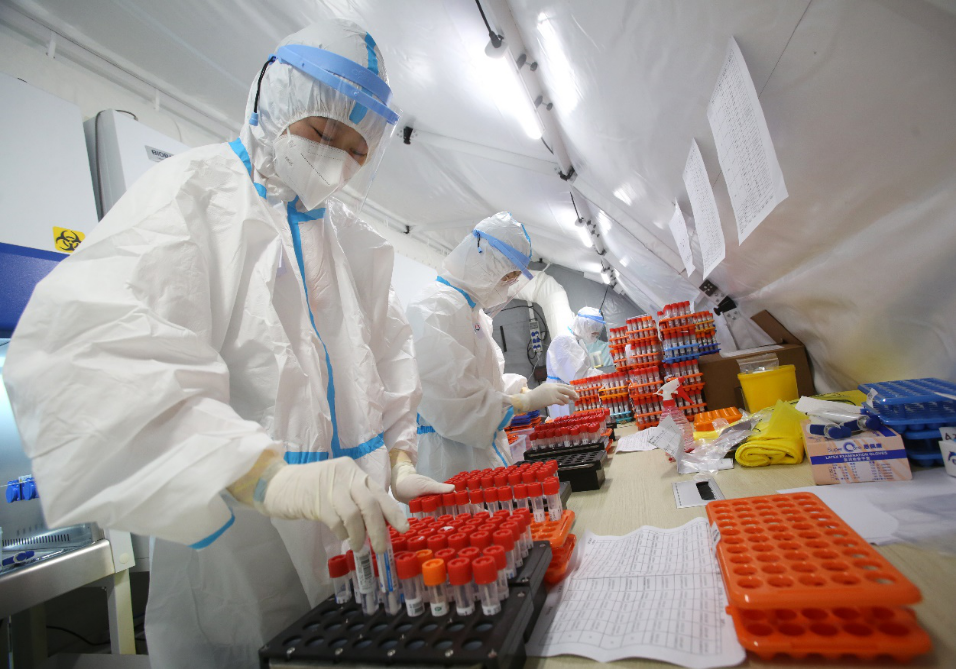
Fig. S4 Photo of makeshift inflatable laboratories**

a, Photo of makeshift inflatable laboratories.

b, Medical staffs and volunteers processed samples in the laboratory.
